# Development of a Digital Decision Support Tool to Aid Participation of Children With Disabilities in Pediatric Rehabilitation Services: Explorative Qualitative Study

**DOI:** 10.2196/14493

**Published:** 2019-10-02

**Authors:** Elin Vinblad, Ingrid Larsson, Maria Lönn, Emma Olsson, Jens M Nygren, Petra Svedberg

**Affiliations:** 1 Child and Youth Rehabilitation Services Region Skåne Kristianstad Sweden; 2 School of Health and Welfare Halmstad University Halmstad Sweden; 3 Region Halland Halmstad Sweden; 4 Falkenbergs Municipality Falkenberg Sweden

**Keywords:** child, child care, decision making, eHealth, disabled children, patient participation, rehabilitation, qualitative research

## Abstract

**Background:**

Building a health care system in accordance with the rule of law requires child-centered care, where children and young people, regardless of ability, are allowed to participate in visits with their health care professionals. As part of an overall project focusing on developing and implementing a digital decision support tool to increase the participation of children with disabilities in pediatric rehabilitation, this study brings new knowledge as to how this specific patient group views participation.

**Objective:**

The aim of this formative study was to explore the experiences of children and young people with disabilities concerning increasing their participation in the pediatric rehabilitation services.

**Methods:**

The formative study had an explorative design, based on a latent qualitative content analysis with an inductive approach. Interviews were conducted with 20 children (6-17 years) and 8 young people (19-30 years) with disabilities about their experiences of participation in pediatric rehabilitation services.

**Results:**

A total of 3 categories emerged reflecting the participants’ possibilities of participation in the pediatric rehabilitation services: to feel involved, to feel independent, and to work in partnership. To feel involved meant being listened to and being connected, to feel independent meant being admitted and being enabled, and to work in partnership meant being supported and being able to entrust others with the decision making. With the overall theme *moving toward empowerment of children in pediatric rehabilitation*, a true feeling of participation can be experienced.

**Conclusions:**

The views of children and young people with disabilities are that children should be given the prerequisites for empowerment by being allowed to feel involved and independent as well as to work in partnership to experience true participation in the pediatric rehabilitation services. This finding is essential in the design of a digital decision support tool based on the children’s needs and perspectives.

## Introduction

### Background

The Patient Act in Sweden [1] aims to protect the right of an individual to participate in health care decisions. Allowing patients to participate in decision making has been shown to improve patient-reported satisfaction, patient compliance, and reported quality of care [2]. The United Nation’s Convention on the Rights of the Child (UNCRC) will be incorporated into Swedish law on January 1, 2020, thus supplementing the Patient Act [3]. The UNCRC states that all children have a right to participate in society, to have an opinion, and to be listened to in all matters affecting them [4]. The incorporation of the UNCRC into Swedish law, together with the Patient Act, further increases the pressure on pediatric rehabilitation services to involve the children in their care, as child participation becomes both a prerequisite for quality health care as well as an obligation by law.

The implementation of interventions that support children’s participation in health care is still rare [5-8] and especially where children with disabilities are concerned [9]. The consequences of unsatisfactory participation are, however, particularly severe for children with disabilities as their needs for extensive care place greater demands on efficient interaction with professionals [10,11]. A core method for strengthening the patient’s active role in health care is *shared decision making* (SDM), where the patient and staff work together to make decisions based on current evidence and the patient’s experiences, while also considering different alternatives, goals, and preferences [12]. Although SDM aligns well with the principles of UNCRC, research has shown that child participation in health care and rehabilitation services is almost nonexistent [6,13,14], thus indicating a lack of strategies to promote child participation. This is true for most children, and especially where children with neuropsychiatric and intellectual disabilities are concerned [11,15].

The pediatric rehabilitation services in Sweden have a tradition of a family-centered approach, where the whole family is regarded as the client. Researchers have in recent years asserted the need for a conceptual move from a family-centered practice toward a child-centered practice within health care services. Taking the concept of child-centered practice one step further, researchers now stress the need to move from earlier guidelines of *child-focused* health care toward *the child’s focus* within their own health care [16]. The importance of including the child´s perspective has been interpreted as the importance of inviting children to share their needs [17-20] and allowing the child to take a more active role in goal formulation and decision making [21,22]. The use of SDM is thus supported as well as promoting the fulfillment of the UNCRC. The child is involved and accepted as a partner in a child-centered way of working, thus strengthening the child and increasing the possibilities for gaining confidence and independence [23]. However, knowledge regarding how to move toward a more child-focused practice is at present scant, and research is thus needed. In a study aiming to identify barriers and facilitators for SDM in mental health services for children and young people, Gondek et al [13] found that SDM is not always consciously practiced, although staff considers that they fulfill the requirements upon learning the definition. The same study also found that, according to the caregivers, young people who are not included in decisions about their care, may be less willing to follow treatment protocols, thus indicating the need to involve children and young people in decisions regarding their health care [13]. It has been found in the literature that children’s involvement in research that concerns them increases the relevance, content, and ethics of the research and results [24,25]. It is, thus, necessary for researchers to include the children that are affected when investigating how the transformation toward a child-centered practice can be achieved. However, it has been identified in the previous research that there are certain difficulties when it comes to including children with disabilities in research. The difficulties may be related to the research setting, such as ethics, having sufficient time and competent interviewers, or related to the child’s specific difficulties, such as language, cognition, and motor skills [23,26-28]. This has resulted in the group being generally unrepresented within research that affects them [23,29], and it is, thus, even more essential to involve this target group in the research.

Although research projects incorporating children with disabilities as partners in research appear to be scarce, previous studies have included children from the other areas of health care. These studies, focusing on increasing child participation in health care, have shown that digital tools providing support for communication and participation have a positive outcome on the children’s involvement and engagement in their own care (IM Carlsson et al, unpublished data, 2019; I Larsson et al, unpublished data, 2019) [30-33]. However, despite widespread optimism about the potential of such digital tools, research reveals that disparities remain in relation to health and well-being among those who are in vulnerable positions such as children with disabilities [34], and the evidence base for informing policy and practice in relation to this is insufficient [35,36].This study is part of an overall research project with the aim of strengthening children's participation in pediatric rehabilitation services by developing, validating, and evaluating an electronic health intervention that is based on a digital decision support tool for children with disabilities. The overall research project involves children with disabilities, their parents, young people with disabilities, and professionals in a user-centered design process, striving to mediate the children’s voices and introducing a child-centered way of working in pediatric rehabilitation services.

### Objectives

The aim of this formative study was to explore the experiences of children and young people with disabilities about increasing their participation in the pediatric rehabilitation services.

## Methods

### Design

This formative study had an explorative design, with the purpose of informing the development of an intervention that is based on a digital decision support tool for children with disabilities. Formative research helps the researcher to identify and understand interests, behaviors, and needs of target populations to inform the decisions and actions when designing and implementing an intervention [37]. The analysis was based on qualitative content analysis, with an inductive approach. Qualitative content analysis can be used to identify similarities and differences in text-bound material [38] and offers a flexible, pragmatic method for developing and expanding knowledge as well as understanding human experiences [38,39]. Qualitative content analysis has been widely used in health care research and was considered an appropriate approach for this study as the target group’s views on participation were assumed to vary greatly.

### Participants

The target group was 28 children and young people with disabilities. The inclusion criteria for the children were as follows: aged 6 to 18 years, with an established contact with the pediatric rehabilitation services in Southern Sweden. Furthermore, the children had to be able to participate in an adapted interview setting, as well as answering questions using their chosen mode of communication. A total of 20 children were included in the study. Most children had been using the rehabilitation services since early childhood, with an average of 6.3 years of contact. For the young people, inclusion criteria were as follows: aged 19 to 30 years, with previous established contact with pediatric rehabilitation services in Southern Sweden. A total of 8 young people were included in the study (Table 1).

**Table 1:** Sociodemographic data (n=28).

Characteristic		Number of participants
**Age range (years)**		
	6-10	8
	11-13	7
	14-17	5
	18-24	6
	25-30	2
**Sex**		
	Female	15
	Male	13
**Main disabilities**		
	Physical disability	14
	Intellectual disability	5
	Autism spectrum disorder	9

### Data Collection

The recruitment of and individual interviews with the children with disabilities were carried out in 2017 and early 2018 by the first author (EV), who was an employee at a local pediatric rehabilitation center in Southern Sweden with an experience of working with children with disabilities. Each child was able to decide when and where the interview was to take place and whether they preferred another adult to attend. Only one child chose to have a parent present. The interviews varied from 20 min to 60 min in length and followed a semistructured interview guide with open-ended questions, resulting in an informal dialogue with the children in a conversational tone. The children were asked to expand certain statements and were guided through their experiences of participation. They were also encouraged to forward thoughts to the researcher that could arise after the interview, an offer which 2 participants used.

The recruitment of the young people with disabilities was carried out in 2017 by an established contact person from a pediatric rehabilitation service in Southern Sweden. The young people first participated in group interviews held by the third, fourth, and last authors (ML, EO, and PS); the group size varied between 2 and 5 people. Individual interviews were then carried out with 2 participants who were considered able to further contribute with essential data [40]. The data collection was carried out in semistructured interviews, starting with open questions such as ‘‘Can you describe what participation in your pediatric rehabilitation looks like to you?” To attain greater depth in the data, follow-up questions were used to further investigate the participants’ views and experiences. Participants had full access to all augmentative tools needed throughout the interviews, and all the interviews were digitally recorded.

### Data Analysis

Qualitative content analysis was used, and both the manifest and latent content was analyzed [38,41]. The interviews were transcribed verbatim by the interviewing authors (EV, ML, and EO) and were read several times to gain familiarity. The first (EV) and second (IL) authors conducted the initial analyses of the children’s interviews, while the initial analysis of the young people’s interviews was carried out by the third and fourth authors (ML and EO). In the initial analysis, meanings or phrases with information relevant to the object of the study were identified and extracted, together with the surrounding text, to preserve the content. The meaning units were then abstracted and coded. Then, the first (EV) and the second (IL) authors searched for differences and similarities in the total material from both children and young people before grouping the codes into subcategories and categories. A total of 6 subcategories and 3 categories, reflecting the core message in the interviews, constituted the manifest content. The content of the categories was abstracted into a theme reflecting the underlying meaning, constituting the latent content. The analysis was discussed in the research group to establish consensus. Representative quotations from the children and young people were used to illustrate the data in the categories. An example of the data analysis is shown in Table 2.

**Table 2:** Examples of meaning units, codes, subcategories, categories, and theme.

Meaning unit	Code	Subcategory	Category	Theme
*I tell them [that I don’t want to] and we do something else.* [Child 9]	The professionals listen to the child.	Being listened to	Feeling involved	Moving toward empowerment of children in pediatric rehabilitation
*But I know it best [what I need].* [Child 6]	The child is respected.	Being connected	Feeling involved	—^a^
*It’s like...I can understand what they are saying*. [Child 4]	The child understands the information.	Being admitted	Feeling independent	—
*To be allowed to take it at your own pace, to be allowed time depending on your challenges.* [Young person 4]	Adjusting the child’s pace.	Being enabled	Feeling independent	—
*When I needed a new chair, dad printed all the papers because he knew we had to convince [the therapists of the need for a new chair].* [Young person 2]	The child has parental support.	Being supported	Working in partnership	—
*I think it’s better if they [the therapists] decide...* [Child 11]	The child trusts adults in decision making.	Entrusting decision making	Working in partnership	—

^a^All the categories were abstracted into the overall theme moving toward empowerment of children in pediatric rehabilitation.

### Ethical Considerations

This study conforms to the ethical principles for research on human beings as set out by the World Medical Association in the declaration of Helsinki [42] as well as the national guidelines on ethical principles [43]. The ethical approval was granted by the Regional Ethical Review Board at Lund University, Sweden (No: 2017/707). The children, young people, and parents received oral and written information about the study and the voluntary nature regarding participation and their right to withdraw at any time without explanation. Written informed consent was given from the parents of participating children as well as from the participating young people before their inclusion in the study. The children gave their consent either in writing or orally. The children and young people were, immediately after the interview, given the opportunity to discuss any emotions or thoughts that had emerged with staff who possessed the necessary knowledge to deal with their concerns.

## Results

The results reflecting the children’s and young people’s positive experiences of participation in the pediatric rehabilitation services included the overall theme *Moving toward empowerment of children in pediatric rehabilitation* and 3 related categories: *Feeling involved*; *Feeling independent*, and *Working in partnership* (Textbox 1).

**Overview of theme, categories, and subcategories.**

Theme: Moving toward empowerment of children in pediatric rehabilitation.

Categories and subcategories:

- Feeling involved
  - Being listened to
  - Being connected
- Feeling independent
  - Being admitted
  - Being enabled
- Working in partnership
  - Being supported
  - Entrusting decision making

### Moving Toward Empowerment of Children in Pediatric Rehabilitation

The children’s possibilities for moving toward empowerment in pediatric rehabilitation incorporated their experiences of participation in each of the categories. When children felt involved, independent, and were in partnership, they learned to trust that their voices would be heard, that they would be granted access, and that they would be able to make decisions or entrust others to make decisions for them. This, in turn, led to inner feelings of inclusion, autonomy, and self-efficacy that boosted empowerment.

#### Feeling Involved

The category *Feeling involved* incorporated the subcategories of *being listened to* and *being connected*. The category emerged from the importance of professionals addressing the child directly, asking questions and listening to the answers, encouraging the child to make suggestions and keeping the child at the center of their attention. When a child was used to feeling involvement, a higher level of self-esteem was developed, which was in turn related to the child’s ability to demand participation for himself. When a child was not included, this led to exclusion, a low level of self-esteem and a lack of confidence in demanding participation.

##### Being Listened To

The subcategory of *being listened to* contained children’s and young people’s experiences of contributing with opinions that would directly affect the care they received as well as taking part in the decision making as compared with only being present at a meeting. Both children and young people could reflect that this is easier for children with a high level of self-esteem as these children are more likely to demand participation. Although a child simply expressed that “you have to be really good at saying what you want,” a young person expressed a deeper understanding that children with varying abilities have varying possibilities of participation:

I have had the chance to discuss [my care], but that’s because I’m an outgoing person as you can hear, and therefore, I have an advantage.

Young person 1

Both children and young people wanted to be listened to and be allowed to take part in the decision making concerning their care and thus feel involved. Regarding the right to make decisions, the young people leaned more toward allowing children to decide everything for themselves, whereas the children preferred to share the responsibilities:

Perhaps children shouldn’t decide everything, but at least a little...So that everyone is happy.

Child 17

Both children and young people expressed an understanding that not everything is optional where necessary procedures regarding their rehabilitation were concerned. They did, however, wish for a child to be involved in briefings about the procedures to answer questions and capture any negative emotions that may arise. A child explains in the following quote why the inclusion of children in decision making can affect their motivation to participate in the care that the professionals deem necessary:

Because it’s all about me. I’m the one who then has to do everything...Otherwise, it won’t turn out as well, and it wouldn’t be the way I want it to be, and then, it won’t be as fun [doing the exercises].

Child 8

A topic that emerged in terms of being listened to, was the right to object to nonvital decisions. Although the young people emphasized the need to listen when a child says no, the children on the other hand simply assumed that their opinions, if expressed, would be respected. Even children who preferred not to be part of the decision making were confident that if they raised any objections to what the adults had decided, the decisions would be revised. A child was asked about what would happen if the child did not approve of the exercises that the therapist had decided:

I tell them that I don’t want to. They’ll listen to me.

Child 10

The young people wished that staff at the rehabilitation center would at an early stage encourage the child to speak its own mind while at the same time requesting the parents to not speak as much. Furthermore, the young people expressed a need for adults to focus completely on the child, and parents who did not cooperate would have to leave the room.

##### Being Connected

The subcategory of *being connected* encompassed a feeling of belonging in the different rehabilitation settings, as well as being respected as an individual. This could mean establishing a positive relationship with one’s caregivers, feeling welcomed and being asked one’s opinion, being familiar with one’s condition, as well as being presented with choices. One child explained why it is important that the doctor not only puts questions to the parents but also to the child:

Because I have everything. Up here [points to forehead].

Child 9

Another child explained that it is important that adults involve children in discussions concerning their diagnosis and prognosis so that the child can not only explain the situation to classmates but also motivate themselves to do necessary exercises:

If I have to do something boring and I don’t even know why I have to do it, then it will be even more boring...I have to understand it so that I’m not just showing up at the hospital and don’t even know why.

Child 8

Both the children and the young people pointed out the need to understand their abilities to be more independent and self-assured. Being able to understand their needs and communicate them helped in feeling connected to the discussions around them:

I needed it on paper, written what I can and cannot handle...Having it on paper like this, I was taken more seriously and I like that.

Young person 4

The young people described that when they were children, they wanted to be involved and accepted as an individual and treated with the same respect as an adult. They also wanted their rehabilitation work to be based on their interests, so that they could be motivated to perform it.

Parents were sometimes described as barriers, in this category, as the professionals might turn directly to the parent instead of the child, thus resulting in the child being excluded and disconnected.

#### Feeling Independent

The category *Feeling independent* concerned the child’s possibilities of accessing the services at the rehabilitation center as independently as possible, both physically and otherwise, which was manifested in the subcategories: *being admitted* and *being enabled.* Unnecessary barriers to participation could be removed by having sufficient access and through adjustments being made, their dependence on adults could be decreased, and the child could focus on the more permanent barriers that came with the disability. Both children and young people expressed ideas relating to developing their independence, thus striving toward adulthood where they would not have to rely on their parents for everything.

##### Being Admitted

When the participants experienced the possibility of being admitted, they described a feeling of being able to commute to the center, being able to get in contact when they need to, and understanding the information they received. Having unhindered access contributed to higher levels of both independence and participation.

The children expressed a strong desire to have access to the rehabilitation services in terms of being able to get in contact with them when they needed to, regardless of which day it was or what the time of day was. They also expressed a desire to be able to contact the services independently of their parents, making suggestions about having a direct chat link to their therapists, or a walkie-talkie mounted on their wheelchairs. The children expressed no concerns regarding weekends or holidays, and they instead appeared to believe that their staff lived at the rehabilitation center and would always be accessible if they only had a way to communicate with them. The young people also preferred a phone call instead of participating in meetings with several unknown adults:

No thank you, I’ll call when I need the help.

Young person 2

Another concern among the young people was that of difficulties visiting the pediatric rehabilitation center and instead wanting to access activities closer to home:

I think, if you live in a smaller town, you should be able to get help there as well.

Young person 2

The young people talked of the distance between their home and the rehabilitation service influencing the help they received and how likely they were to request the help that could be supplied. The sense of there being long distances between the school, the pediatric rehabilitation services, the family, and the child was experienced as a barrier for the child’s participation. In contrast with the young people, the children expressed beliefs that adults could simply make the logistics work.

##### Being Enabled

The subcategory of *being enabled* included experiences of having both physical and cognitive adjustments to be able to participate independently. Participants in wheelchairs expressed a great need for adjustments to freely experience their environment:

Sometimes I need help in controlling [the environment]...Like moving things, so I can get through with this one [points to wheelchair].

Child 1

Both children and young people were satisfied with their physical surroundings at the rehabilitation center, instead of focusing on cognitive adjustments to gain enablement. Adapted information was considered important by both children and young people. The children talked about how adults like to sit in meetings and talk, whereas they themselves preferred not to participate. Some children, but not all, preferred to have a child-adapted premeeting with only 1 member of the staff, where the child could communicate its needs more comfortably. In the quotation below, the child has explained that it is easier to talk to just 1 person and says how this usually works:

We plan together what they [the adults] should talk about next time. And then we have decided, she and I.

Child 1

Children and young people knew that complex matters need to be discussed sometimes and then emphasized the need for adjustments to enable the child’s understanding of the matter. Being enabled to comprehend their rehabilitation increased the experience of independence. The young person in the quotation below explained how understanding the information helped increase participation while at the pediatric rehabilitation center:

They sat down and took their time to explain so that I could understand based on my prerequisites.

Young person 3

In the quote mentioned above, the young person mentions the importance of having enough time to comprehend. This thought was common among the young people, who expressed a need to have sufficient time with the professionals, otherwise, they felt that the meeting was stressful and would rather not participate in the rehabilitation work at all. This idea was quite the opposite among the children, who did not want their encounters with adults to be too long, or they would tire and lose their motivation.

#### Working in Partnership

The category *Working in partnership* captured the different enablers in the child’s surrounding network who worked together with the child to increase his/her level of participation. Working in partnership included the subcategories of *being supported* and *entrusting decision making*. The facilitating partner was described as an adult who enabled the child’s participation by bridging the gap between the child and the professionals, as well as an adult who could speak on behalf of the child when the child felt the need for it. The children wanted to work in partnership with adults, not in terms of receiving support based on the adults’ perspectives, but rather based on what the children themselves define as important.

##### Being Supported

Both children and young people expressed similar opinions regarding the appreciation of good support from adults. If the children were put in a situation that they did not enjoy and were not capable of telling the staff, they leaned on parents to communicate for them. Not all children were comfortable expressing their opinions, explaining that they needed support to find courage:

My counselor tells me that I must be brave...I have some problems with talking about my emotions and needs at home, but I can talk to my counselor.

Child 13

The young people described how parents were necessary to support them when they did not have the energy to participate themselves, or when they did not understand the discussions about their care:

Sometimes the rehabilitation doctor asks questions that can be difficult for children to even understand...and then I look at them and ask “what are you talking about?”...Sometimes I want my parents to join me so that they can interpret and explain.

Young person 3

The young people could also reflect on the need for 1 adult to take charge of the cooperation between all the adults around the child so that the child did not have to take responsibility for keeping everyone informed. This reduced the pressure on the child, leaving him/her free to focus on other things. One young person explained that a child might sometimes also need direct support in handling all the contacts:

Like if you’ve got lower intelligence or difficulties coping with many things at the same time; then it’ll all be too much...When you have to check this thing, and that thing, and call this one and then call that one...You probably need some extra help there.

Young person 1

##### Entrusting Decision Making

The category of working in partnership also captured a more indirect version of participation, presented in the subcategory of *entrusting decision making*. Children generally wanted to be given a choice of when to participate and when to delegate certain decisions to others whom they trusted. When asked to elaborate, the children replied that they are content with deciding some things, but not other things. The following child was asked about whether the therapists gave him enough space to decide about his individual gym practice:

I only want to decide about the football [the chosen reward after gym practice].

Child 5

Another child expressed a deep trust in the therapists and a desire not to intervene:

I think it’s better if they [the therapists] decide...Because she does good things. So I think I should do what she says.

Child 11

The children seemed to think that the act of entrusting the decision making to a partner could both serve as a way to save their energy and leaving them free to focus on more pressing matters, as well as ensuring high-quality health care by allowing the professionals to determine the direction of the therapy. The young people, on the other hand, did not express any wish to pass on the act of decision making to others, but could see positive gains in having a trusted adult speak on their behalf:

Perhaps if there could have been a contact person there instead, who could pass it on [my needs and wishes], instead of me sitting there with a bunch of curious old ladies.

Young person 2

Having adult partners such as these, who could make decisions on behalf of the children or pass on important information, meant the children could participate on their own terms. By choosing when, where, and how to participate, they could adapt their level of inclusion to suit their current situation.

## Discussion

### Principal Findings

This study is part of an overall research project aimed at developing, implementing, and evaluating a digital decision support tool to increase participation for children in pediatric rehabilitation. The result from this formative study will further develop the forthcoming digital tool by contributing with ideas that can promote participation according to the target group themselves. The aim of this study was, thus, to explore the experiences of children and young people with disabilities about increasing their participation in the pediatric rehabilitation services. The participants define participation from their own point of view, resulting in the categories of feeling involved, feeling independent, and working in partnership. Combining the categories lead to the identified theme *moving toward empowerment in pediatric rehabilitation.* When a child is able to participate in a meeting where the child feels listened to, understands the conversation, and is able to choose whether to take part in the decision making or not, the whole meeting becomes centered around that child, where he/she is always able to interject if he/she wanted to. This, in turn, can increase the child’s self-confidence and can be assumed to increase the chances that the child will want to claim the same rights at the next appointment at the pediatric rehabilitation center. This can be surmised as being able to lead to individuals being empowered and aware of their rights for inclusion and autonomy. The results thus indicate that the digital decision support tool can support children’s self-efficacy and autonomy to create greater possibilities for participation in rehabilitation services. The digital communication tool needs to be constructed in such a way to meet these children’s developmental, intellectual. and cognitive levels, so that they have the capacity to obtain and understand the information needed to participate in their own care. The desire to feel independent, involved, and being able to work in partnership with adults needs to be addressed and incorporated into the digital tool—a solution that will be covered in future studies. An aspect of particular interest, which would not have been considered without the input from the children, is the need to be able to consciously delegate decisions.

The results reveal that children and young people with disabilities experience participation through feelings of involvement. The participants in the present study express an awareness of the fact that involvement may be difficult to achieve, addressing matters relating both to their own person and to their surroundings. Participants who express a high level of belief in themselves also express greater independence and a willingness to take more responsibility in their rehabilitation, thus demanding to be included and involved. This relates to research stating that a person’s internal characteristics such as capacity, ability, self-esteem, self-efficacy, and motivation are affected by previous experiences, which in turn influence future behavior, performance, and experiences of participation [11,19,44,45]. Research also concludes that relational factors such as effective communication between patients and staff is crucial for high-quality health care for people with disabilities [46,47] and that low communication skills negatively affect the possibilities for participation [48]. In this study, both children and young people with disabilities indicate the need for easier ways to communicate with professionals, whereas also reflecting that there are great demands on children to be forthright. This supports the concept of a decision support tool with simplified modes of communication that can be used to increase the child’s possibilities of being involved and active in their rehabilitation, where the children feel that they are respected and that they are able to be part of the decisions concerning their care.

The children and young people reveal a high dependence on being able to access the rehabilitation facilities. Research indicates that the location of health care services impacts greatly on the patients’ likelihood of attending [47] and should be considered an important factor regarding children with disabilities, who are very dependent on adults providing transportation. Although the concept of being independent can be linked to broader discussions of autonomy, the participants only expressed concerns regarding physical and cognitive access, indicating that these issues are more pressing. If these obstacles were more satisfactorily solved, the participant could perhaps be able to consider autonomy in a broader sense of the word. Having cognitive access, that is, being able to understand the information, is viewed as an important factor for the participants. Some appreciated being allowed enough time to comprehend, whereas others wished for shorter meetings so as not to lose interest, thus once more emphasizing the need for a child-centered health care that acknowledges the child’s focus [16]. Considerations have to be made concerning the dual nature of the findings in the research literature in terms of the possibilities for understanding the interactions with professionals. There is a need for simplified communication to avoid health care barriers [47], and recognition of people with disabilities often feeling belittled by caregivers’ inappropriate assumptions regarding their low levels of understanding [48]. It is important to carefully consider the individual needs for either a more simplified information or a more advanced exchange in the development of this digital decision support tool. The results suggested that a digital decision support tool needs to provide an individualized format that supports the children’s possibilities for communicating their own perspectives of their situation and health. Furthermore, a digital tool that helps children to report their needs, problems, strengths, and experiences, could also be beneficial for staff in understanding the unique child’s situation and provide a more child-centered rehabilitation. Greater consideration needs to be taken of individual experiences, and professionals need to consider that factors such as individual experiences cannot be objectively observed.

The results highlighted both the children’s and the young people’s appreciation of good support from and a partnership with adults. The term *gatekeeper* is used in the previous research to describe a person close to the child who can both facilitate and hinder the child’s involvement in health care [23]. Participants agree that parents can serve as both facilitators and barriers depending on the setting and how the health care professionals treat the child in relation to the parent. The need to be seen as a partner in decision making is evident, where the child can choose to participate among other equals or to step aside knowingly trusting others. However, the children’s desires to be able to pass on certain decisions to adults, a thought that the young people do not seem to share, is a desire that needs to be carefully dealt with. It can on the one hand be a highly autonomous decision to be left out of the decision making, for example because of energy-saving concerns, but on the other hand it can represent the beginning of a pattern of low participation. It has been claimed in some previous research that participation competence can be actively trained and developed [49] but it seems reasonable to reverse the equation: If a child repeatedly wishes to entrust adults with their decision making, a passive attitude toward participation could be manifested. Research also states that each situation of delegation requires careful considerations, relating to the patient’s unique needs and preferences [50], thereby motivating the need for the future digital decision support tool that can provide possibilities for delegating the decision making while also urging the staff to be mindful of this request. It is, however, important to point out that participation is a right and not an obligation. It, thus, becomes important in the forthcoming digital tool for children to be able to choose which questions to take part in and which to entrust to others.

### Methodological Considerations and Limitations

The interviews were conducted both individually and in pairs in this study to allow different settings for different needs, as recommended by Krol et al [20]. The individual interviews generated a profundity and became more personal, whereas group and pair interviews have the advantage of creating security in the group and giving a voice to those who cannot participate in an individual interview. The different interview forms had the purpose of facilitating the participation of all of the participants. As children can be hesitant to speak in very formal settings [51], allowing them to participate in a well-adapted situation contributes to securing their views and opinions in the research. Depending on each participant’s specific difficulties, the interviewer also had to consider potential challenges in terms of social skills, cognition, language skills, memory, as well as concentration and endurance. This, thus, entails a great demand on the interviewer to present appropriate questions with sufficient communication aids [26,27], using augmentative tools [28] as well as adapting the setting based on individual needs. The adaptations needed to complete the interviews also gave an insight into which adaptations can be needed in the digital decision support tool, such as having communication supported by pictures and manual signs.

This formative approach, with a constant seeking for the individual child’s perspective, can help contribute to a tool that can better capture the children’s experiences and needs. The children and young people in this study are included as partners in the research, where they evaluate certain aspects of their life situations themselves, without input from professionals. Previous studies have pointed out the need to include children in the research that affect them [30] as well as the need to include children with disabilities as partners in the research [23]. To our knowledge, there has been very little research that combines the 2: the inclusion of children with disabilities in research that is primarily aimed at improving their situation. A further novel aspect is the concept of including children with disabilities in research to improve their participation, and as cocreators of a digital decision support tool. We have not found any studies that have put this to practice, thus making the results presented in this study unique.

A weakness of this study, as with all retrospective studies, is that the young people look back at previous experiences that may not represent the current way of working in the pediatric rehabilitation services. The children, however, present their current views and provide statements that are highly important for the services to access. Nevertheless, all children have the right to participate and a need to be listened to which is confirmed in this study as well in others [13,18].

### Conclusions

This formative study explored children’s and young people’s experiences of participation-enhancing factors in the pediatric rehabilitation services, with the aim of incorporating their experiences in the development of a digital decision support tool for increased participation. The findings concluded that children experienced participation through feeling involved, feeling independent, and working in partnership. When these requirements were met, the children were able to move toward empowerment in their pediatric rehabilitation. Experiencing participation through involvement meant feeling listened to and feeling connected, feeling independent meant being admitted and being enabled, and working in partnership meant feeling supported and being able to entrust the decision making to others. This definition of participation belongs to the participants themselves and can only to some extent be compared with other research. The participants’ opinions are core elements when developing an intervention with a digital decision tool to support the children’s participation, and their contribution will help in making the tool attractive, relevant, and in line with the UNCRC. However, to safeguard children’s right to participation, future research must explore potential obstacles to participation as identified by the target group themselves.

## Abbreviations

SDM: shared decision making
UNCRC: United Nation’s Convention on the Rights of the Child
